# Improving Retrieval Augmented Generation for Health Care by Fine-Tuning Clinical Embedding Models: Development and Evaluation Study

**DOI:** 10.2196/82997

**Published:** 2026-03-25

**Authors:** Kamyar Arzideh, Henning Schäfer, Ahmad Idrissi-Yaghir, Cynthia Sabrina Schmidt, Bahadir Eryilmaz, Mikel Bahn, Amin T Turki, Olivia Barbara Pollok, Eva Maria Hartmann, Philipp Winnekens, Katarzyna Borys, Johannes Haubold, Felix Nensa, René Hosch

**Affiliations:** 1 Institute for Artificial Intelligence in Medicine,, University Hospital Essen, Essen, Germany; 2 Central IT Department, Data Integration Center, University Hospital Essen, Girardetstr. 2, Essen, 45131, Germany, 49 0231-77816; 3 Institute for Transfusion Medicine, University Hospital Essen, Essen, Germany; 4 Institute of Diagnostic and Interventional Radiology and Neuroradiology, University Hospital Essen, Essen, Germany; 5 Center of Sleep and Telemedicine, University Hospital Essen, Ruhrlandklinik, Essen, Germany; 6 Department of Hematology and Oncology, Ruhr-University Bochum, Marienhospital University Hospital, Bochum, Germany

**Keywords:** Retrieval Augmented Generation, RAG, natural language processing, information retrieval, large language models, LLM, NLP

## Abstract

**Background:**

Embedding models are critical components of Retrieval Augmented Generation (RAG) systems for retrieving and searching unstructured medical data. However, existing models are predominantly trained on publicly available English datasets, limiting their effectiveness in non-English health care settings. More importantly, these models lack training on real-world clinical documents, leading to inaccurate context retrieval when integrated into RAG systems for health care applications. This gap is particularly pronounced in specialized medical documentation containing domain-specific terminology, abbreviations, and nuanced clinical language.

**Objective:**

This retrospective study aimed to develop and validate embedding models specifically trained on real-world clinical documents from multiple medical specialties to improve medical information retrieval (IR) and RAG system performance in both German and English language contexts.

**Methods:**

We fine-tuned embedding models, so-called sentence transformers, using the multilingual-e5-large architecture as a foundation. Training data consisted of approximately 11 million question-answer pairs synthetically generated from 400,000 diverse clinical documents from a large German tertiary hospital, spanning 163,840 patients and 282,728 clinical cases between 2018 and 2023. The *SauerkrautLM-SOLAR-Instruct* large language model generated medically relevant questions and corresponding answers for each document. The dataset was additionally pseudonymized and translated into English to aim for broader applicability. Models were evaluated in 2 distinct scenarios: IR using questions with multiple relevant passages, and RAG system performance in both cross-patient and patient-centered contexts.

**Results:**

In the IR evaluation, the fine-tuned miracle model achieved a mAP@100 of 0.27, outperforming the multilingual-e5-large baseline (0.14) and state-of-the-art models such as bge-m3 (0.11). In the RAG evaluation, the model demonstrated robust performance comparable with the baseline in the constrained patient-centered scenario (BERTScore F1 0.781 vs 0.778) and showed moderate improvements in the unconstrained cross-patient setting (BLEURT 0.56 vs 0.53). Notably, the model trained on pseudonymized data achieved comparable retrieval performance (mAP@100 0.25) and the highest scores for patient-centered contextual precision (0.93). Performance gains were robust in the German dataset, while the translated English model demonstrated promising results as a proof of concept for cross-lingual transfer.

**Conclusions:**

By leveraging a comprehensive real-world dataset spanning multiple medical specialties and using large language models for synthetic question generation, we successfully created and validated domain-specific embedding models. These models can improve medical IR in large-scale search spaces and perform competitively in constrained RAG applications. By publishing the models trained on pseudonymized data, other health care institutions can integrate or adapt these embedding models to their needs. This work establishes a reproducible framework for developing domain-specific clinical embedding models, with the potential to improve data retrieval in medical settings.

## Introduction

Integrating natural language processing (NLP) into health care has opened new application areas to improve clinical decision-making and patient care [1-4]. Central to this advancement was the development of language and domain-specific language models [5-9], which are important for accurately accessing and understanding medical documents. In the field of medicine, however, several obstacles have so far limited the applicability of these new tools. First, medical language [10,11] is complex and documentation characterized by specialized terminology, jargon, and abbreviations [12,13], representing a significant challenge for language models in the health care domain. To address this challenge, it is essential to design models that are specifically tailored to the health care sector. Hence, NLP models, mainly developed and trained on publicly available English datasets, fall short in understanding and processing natural language in non-English linguistic contexts [14,15]. Second, medical documents are characterized by a high degree of specificity and contextual nuances, which the existing general language models often fail to capture [16-18]. Consequently, the absence of domain and language-specific models has proven to be a substantial obstacle [19-21] for an effective implementation of artificial intelligence and NLP technologies.

Embedding models learn to represent words or phrases as mathematical vectors, capturing their meaning based on the context in which they appear, making them especially valuable in fields where terms can have different meanings depending on context. Integration of these models into Retrieval Augmented Generation (RAG) systems marks an advancement in information retrieval (IR). RAG may enhance the performance of large language models (LLMs) by combining them with an existing knowledge base or document repository [22]. Typically, RAG is implemented by transforming documents or records into vectors. In response to a specific query, the most similar documents are retrieved and then incorporated into the LLM prompt. They combine retrieval-based and generative NLP models and are heavily reliant on the quality of underlying embedding models for their effectiveness [23]. However, these embedding models, also known as sentence transformers, are primarily trained on publicly available datasets. Using these models in a hospital context with routine documents is potentially difficult as these models have not been trained on real-world unstructured clinical texts. Integrating these models into RAG systems to search through patient documents could lead to undesired or incorrect results. This affects the overall adaptation of RAG systems in clinical practice and highlights the importance of domain fine-tuning.

The development of domain-specific embedding models has the potential to improve RAG systems in the clinical context. By providing more accurate, reliable, and context-aware interpretations of medical texts, such models may improve the information basis for medical decision-making and, via decision support systems, ultimately improve patient outcomes. Given the relevant limitations of existing models, the objective of this study was to develop medical embedding models, exemplarily both in German and English. These models were trained on a large corpus of real-world clinical documents of a large tertiary hospital. This domain-specific training should enhance the performance of RAG systems for patient-related questions. By adapting the models to the medical field, we aimed to enhance their capability to understand hospital jargon and interpret the complex semantic relationships and clinical contexts in medical documentation.

## Methods

### Ethical Considerations

This study was approved by the ethics committee of the Medical Faculty of the University of Duisburg-Essen (approval number 23‐11557-BO). Due to the study’s retrospective nature, the requirement of written informed consent was waived by the ethics committee of the Medical Faculty of the University of Duisburg-Essen. All methods were carried out in accordance with relevant guidelines and regulations and the Declaration of Helsinki. No identifying information or images of individual participants are included in this manuscript or its supplementary materials. Participants did not receive any compensation.

### Data Definition

Our study used a carefully curated dataset comprising a balanced collection of German language clinical notes documented at the University Hospital Essen. The dataset included 100,000 documents from each of the following 4 medical documentation categories: radiology reports, discharge letters, pathology reports, and surgical operation notes. This diverse composition ensures a comprehensive representation of clinical language and terminology. The clinical notes were documented between 2018 and 2023 in the clinical information system of the investigating site and contain information related to the medical history of patients, including diagnostic data and treatment documentation.

The total dataset consisted of 400,000 clinical documents from 163,840 patients and 282,728 clinical cases, resulting in a total number of 163,479,394 tokens, of which 1,541,389 were unique, reflecting the depth and complexity of the vocabulary present in the dataset. Further statistical details, including average document length, token frequency distribution, and other relevant metrics, are summarized for each document type in Table 1.

**Table 1.** Dataset characteristics^a^.

Dataset	Total number of tokens	Total number of unique tokens	Total number of sentences	Average tokens per document	Average sentence length
Discharge letters	86,512,538	901,771	4,406,969	865.13	19.63
Pathology reports	22,313,259	333,028	1,901,434	223.13	11.73
Radiology reports	15,565,379	307,141	1,202,033	155.65	12.95
Operation notes	39,088,218	467,116	3,061,313	390.88	12.77
Total	163,479,394	1,541,389	10,571,749	408.70	15.46

a Displayed are quantitative measures for length and average length across the datasets. “Total number of tokens” was calculated by counting the number of tokens or words in the documents, including punctuation. The lemma form of each token was created and the number of lemmas was used as an approximation for the “total number of unique tokens.” “Total number of sentences” represents the count of all sentences per document. “Average tokens per document” was determined by dividing the total number of tokens by the total number of documents. “Average sentence length” was calculated by dividing the number of tokens by the total number of sentences.

### Dataset Generation

The dataset containing 400,000 documents was segmented into smaller chunks using a recursive text splitting approach implemented in the Python (Python Software Foundation) package *langchain* with a maximum size of 450 characters and an overlap of 80 characters. This overlap was selected to ensure semantic continuity at text segment boundaries, accepting a minor degree of redundancy to prevent loss of clinical context at split points. The maximum character length was informed by the specific structural characteristics of the documents. Clinical information is typically recorded in concise, information-dense paragraphs or itemized lists rather than long-form narrative prose. This splitter uses a hierarchical approach to preserve semantic boundaries by attempting to split text at natural break points in the following priority order: paragraph breaks (\n\n), line breaks (\n), word boundaries (spaces), and finally individual characters. This methodology ensures that complete sentences and paragraphs are preserved whenever possible, only breaking midsentence when individual sentences exceed the 450-character limit. The chunking process resulted in 3,164,164 chunks across all document types.

These chunks were given as input to the *SauerkrautLM-SOLAR-Instruct* model, which has 10.7B parameters and was trained on German translated data. For each chunk, this LLM was tasked with generating 5 medically relevant questions alongside the correct answer that is contained in the document chunk, with the intention of simulating a real-world clinical questioning scenario. The model was deployed within the secured clinical network in order to ensure data protection for patients and physicians.

**Figure 1.**
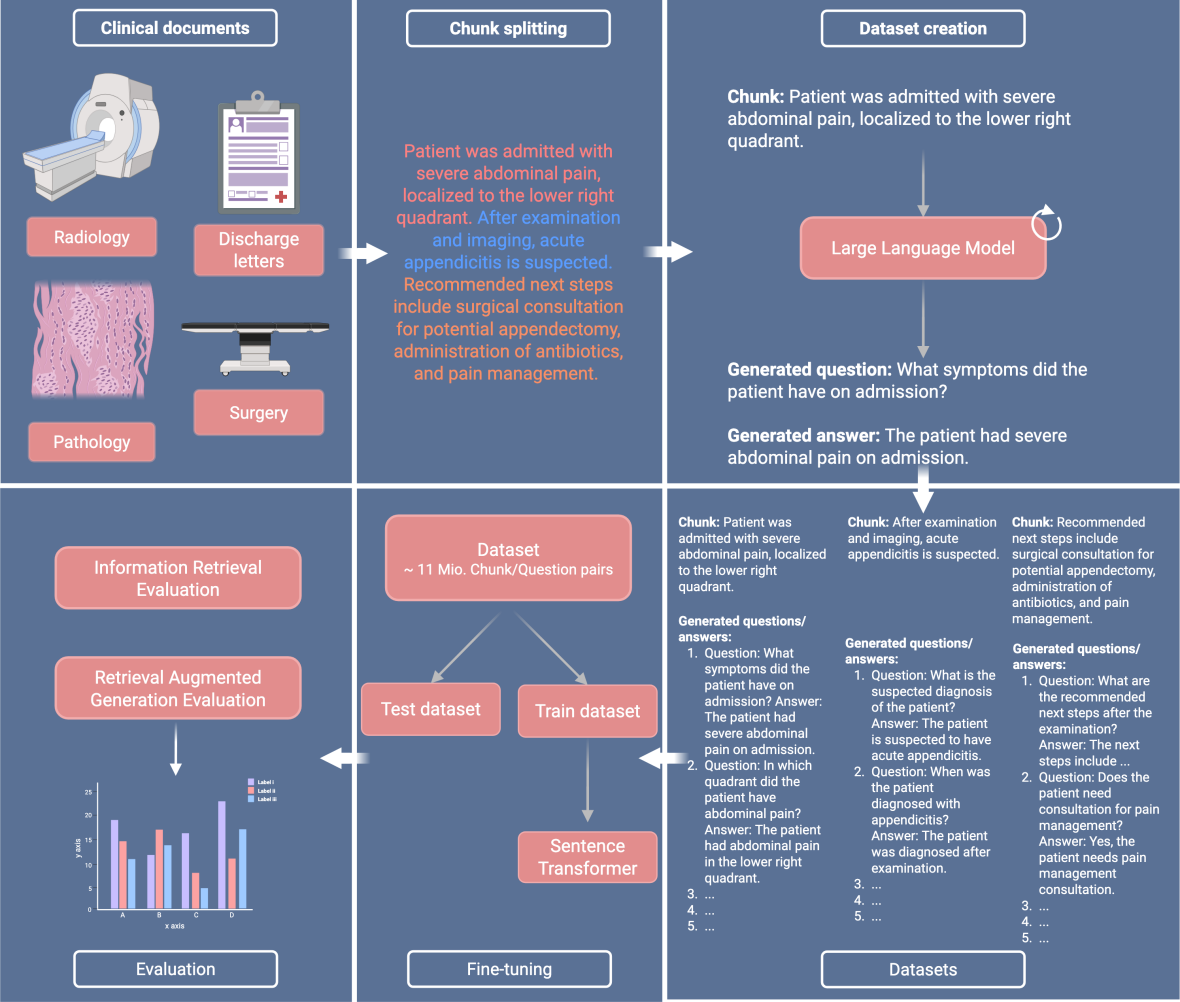
Overview of training and evaluation methods**.** For each text chunk, 5 question-answer pairs were generated with a large language model. The question and chunk pairs were then used to fine-tune sentence transformer models. Different evaluation scenarios were set up to measure the performance of the models. This graphic was created in BioRender.

This process was applied across all collected documents—radiology reports, discharge letters, pathology reports, and operation notes. As a result, a dataset comprising 15,820,820 medical question-answer pairs was created as a training basis for the LLM. This method was chosen to capture the essence of clinical language and reflect the practical, inquiry-driven nature of medical diagnostics and patient care. To reduce the occurrence of hallucinations associated with the LLM and to enhance the data quality, the question-and-answer pairs were systematically filtered through the application of defined criteria. All questions were required to conclude with a question mark and to start with a number between 1 and 5, ensuring the generation of exactly 5 questions for each chunk. After this process, 11,000,227 questions-answer pairs were used for further training and evaluation steps. An overview of the methodologies including sample questions and answers is shown in Figure 1.

### Dataset Pseudonymization

As the generated dataset comprises personally identifiable data belonging to patients and health care personnel, it underwent a pseudonymization process using a deidentification pipeline developed at the investigating site [24]. Protected Health Information in the documents was identified and replaced with surrogates. All training and evaluation procedures were additionally conducted on pseudonymized documents, and performance was compared with the model fine-tuned on real-world data.

### Dataset Translation

The language used in the clinical documents is German, which limits the applicability of the models to German-speaking hospitals. To overcome this limitation, the dataset was translated into English using the Fairseq WMT’19 German-to-English translation model [25]. Although translating medical content can be complex and may result in inaccuracies due to the specialized terminologies involved, the translation process serves as a method for augmenting the corpus and increasing its accessibility. The decision to use this particular translation model was informed by a head-to-head translation model comparison experiment, including physician’s evaluation. The physician was presented with 30 samples produced by various translation models, including WMT19-de-en, M2M-100 [26], NLLB [27], T5 [28], MBart-50 [29], and OPUS-MT-de-en [30]. The feedback on the medical accuracy and readability of the translations informed the final selection of the WMT’19 model for this task.

### Model Training

Fine-tuning was performed using pairs of real document passages and the generated questions described in the “Dataset Generation” subsection. The data were split 80%/20% into a training and a test dataset on a patient basis so that a patient’s data were part of either the training or the test dataset. This resulted in 8,801,525 chunk-and-question pairs for training and 2,198,702 pairs for testing. Hyperparameters used during training include an initial learning rate of 2e-5 and a batch size of 1024. For learning rate optimization, the AdamW optimizer was used. As a loss function, the *CachedMultipleNegativesRankingLoss* from the sentence transformer library [31] with a minibatch size of 32 was used. To normalize the embedding size, pooling was used to create fixed-sized sentence embeddings of 1024 dimensions. Each training run was performed on 1 NVIDIA H100 80GB graphical processing unit for approximately 8 days.

Training was limited to a single epoch. Given the size of the dataset and the synthetic nature of the training data, this approach was selected to prevent overfitting to specific linguistic patterns generated by the LLM. Additionally, training runs showed that training loss plateaued after 1 training epoch.

### Evaluation

#### Overview

Different techniques were used to measure the performance of the fine-tuned models. The models were evaluated on different datasets across 2 distinct evaluation settings—IR and RAG. The reason for these different evaluation scenarios is the difficulty of measuring the performance of sentence transformer models in a single setup. As there is no single scoring scheme that can give an estimate of final performance, different metrics were used, each giving an indication of performance for each individual task. More detailed descriptions about the exhibited metrics can be found in MultimediaAppendices 1  2.

#### Evaluation Setups

IR evaluation was performed to consider various passages and should give a statement about the model’s ability to find several suitable passages for a given query. The RAG evaluation results should indicate whether the embedding model also improved the response quality in an RAG system and, therefore, benefits context retrieval in clinical RAG scenarios. Together, these 2 setups provide a comprehensive view of the fine-tuned models’ performance compared with non–fine-tuned models. Across all evaluation scenarios, we assessed whether fine-tuning led to measurable improvements on each specific task.

For each evaluation scenario, the models were evaluated on the German or English dataset. Thus, the datasets for each evaluation scenario were also translated from German into English. For the evaluation, the fine-tuned model is referred to as *miracle*, the model fine-tuned on pseudonymized data is referred to as *miracle-pseudonymized,* and the model fine-tuned on pseudonymized and English data is referred to as *miracle-pseudonymized-translated*.

#### Evaluated Models

For comparison of fine-tuned to existing models, 3 publicly available models were evaluated. The first model was the *multilingual-e5-large* sentence transformer [32], which was used as a base model for further fine-tuning. This model was continually trained on a mixture of multilingual datasets and supports up to 100 languages, including German. The second model was *bge-m5* [33], a state-of-the-art embedding model that is able to process inputs of sizes, spanning from short sentences to long documents of up to 8192 tokens. It was also trained on multilingual datasets. The third evaluated model was *gte-multilingual-*base [34] with support for more than 70 languages text lengths up to 8192 tokens. The fourth evaluated model was *German-RAG-BGE-M3*, which is a fine-tuned variant of the *bge-m3* model explicitly trained on German data for document retrieval. All evaluated models, including parameter sizes and links to the model cards on the huggingface hub, are listed in Multimedia Appendix 3.

#### IR Evaluation Setup

The IR evaluation was set up by identifying duplicate questions in the test dataset. These duplicate questions are of particular interest as they allow for 1 question to have multiple document passages that have a positive relation to this question. This can be used for an IR task such as search engines, where typically 1 query has multiple possible search results. In total, 109,795 unique questions and 173,933 document chunks from 52,434 documents and 26,358 patients were used for IR evaluation. On average, each question has approximately 5 document chunks with a positive relation.

The quality of the returned results or passages can be measured by metrics such as *Accuracy@K*, Precision*@K*, and *Recall@K* [35]. For IR systems such as search engines, the problem can be defined as a 2-fold task involving the definition of a search query and multiple results for this query. Using a cutoff threshold K and calculating an evaluation metric within the elements of this threshold can determine the quality of the results. More details about IR metrics such as mean reciprocal rank, normalized discounted cumulative gain (NDCG), and mean average precision (mAP) are provided in Multimedia Appendix 4.

For the IR evaluation, *Precision@K*, *Recall@K*, *MRR@K*, *NDCG@K*, and *mP@K* were calculated by retrieving top-k most similar documents for the given query in the dataset. The retrieved list was then compared to the ground truth. Similar document passages were retrieved using the cosine similarity between a query and top-k passages. For the evaluation, the results of the metrics are displayed for a K value of 10. For mAP, the results for a K value of 100 are shown.

#### RAG Evaluation Setup

**Figure 2.**
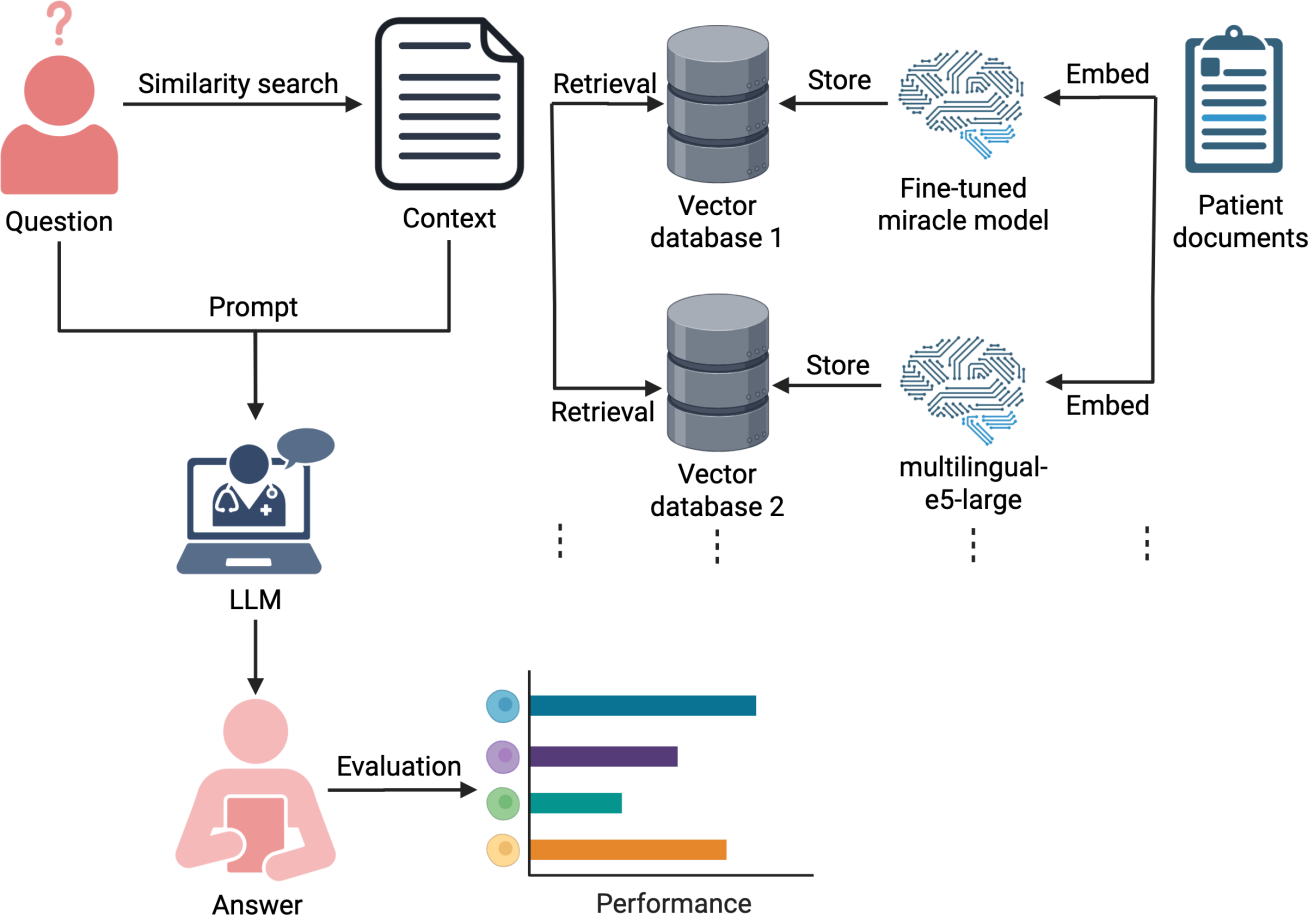
Visualization of Retrieval Augmented Generation (RAG) evaluation setup. For the RAG evaluation, different databases containing embeddings from the different models were initialized. Different RAG systems using these databases as retrieval options were set up, and the same questions were asked of these systems. The generated answers from the RAG systems were compared against the ground truth answer. This graphic was created in BioRender. LLM: large language model.

In order to evaluate the benefit of our fine-tuned clinical embedding model in an IR setting, an experiment was conducted by setting up RAG systems. In Figure 2, the RAG evaluation setup is visualized.

This system received user questions, attempted to find relevant document passages, and provided these passages to an LLM which was prompted to answer these questions. Each question in this dataset was presented to the RAG system and evaluated against the ground truth answer. Different metrics were used to compare the output from LLMs to a ground truth such as ROUGE or BLEURT. Additionally, retrieval-specific metrics such as Contextual Precision, Contextual Recall, and Contextual Relevancy were calculated using the *deepeval* Python package (version 3.7.5). These metrics are based on the LLM-as-a-judge [36] method, using an LLM to assess the quality of the retrieved context and measuring whether the context is relevant to the asked question. For the RAG evaluation, the *gpp-oss-120b* model [37] was deployed and used as the LLM-as-a-judge model. Selected for its high parameter count (120B) and strong performance on reasoning benchmarks, this model serves as an automated evaluator for semantic coherence and relevance, offering a reproducible open-source alternative to proprietary application programming interface–based judges. Detailed definition and explanation of all exhibited metrics are provided in Multimedia Appendix 5.

The fine-tuned embedding model was integrated into the retrieval component of this system, and the results were compared with those of the non–fine-tuned model. Given that the RAG system was identical for both the fine-tuned and non–fine-tuned embedding models, except for the embedding model itself, it was assumed that the greater the number of questions answered correctly, the more accurately the clinical context was identified by the embedding model. For the LLM component of the RAG system, the *SauerkrautLM-SOLAR-Instruct* model was used to answer the provided questions. Since this model was also used to generate the training data for the fine-tuned models, there is a possibility of bias when integrated into the RAG system. Therefore, the *Qwen3-235B-A22B-Instruct-2507-FP8* model was additionally used for generating answers. The maximum number of chunks retrieved for each question was set to 3. Cosine similarity was used as a distance metric for comparing questions and chunks in the similarity search. The smaller this distance, the more similar the question and chunk were considered to be. For easier assessment of the evaluation, Multimedia Appendix 6 shows a prompt example that was used to evaluate answers generated from the LLM using the RAG technique.

The RAG evaluation setup was divided into 2 parts. For the first part, the documents that the RAG system can retrieve were not restricted. This means that for 1 question, document parts can be retrieved from several patients in order to answer the question. While this indicates performance for IR settings, this constellation would rarely occur in real-world scenarios. Therefore, an additional filter mechanism was introduced for the second part to ensure that only documents from 1 patient can be retrieved for each question. Both configurations are referred to as cross-patient (first part) and patient-centered evaluation (second part) in the following sections.

##### Cross-Patient Setup

To prepare a dataset for the cross-patient RAG evaluation, 372 clinical documents from 1 patient each were split into 1129 document chunks. These patients were not included in the training or test dataset that was used to fine-tune the sentence transformer model. The SauerkrautLM-SOLAR-Instruct model was then asked to generate questions and corresponding answers based on the contextual information provided by the chunks. As a result, 5556 synthetic question-answer pairs were generated. These question-answer pairs were filtered by a physician to provide a realistic clinical scenario and human-assisted evaluation. The physician was instructed to remove any questions related to administrative data (eg, questions about the patient’s birth date or names of relatives) or remove questions that had wrong corresponding answers. It was emphasized that only those questions that would be posed in a practical clinical setting and that were factually and semantically accurate should be included. After filtering, 3536 annotated question-answer pairs from 280 documents split into 890 chunks remained for evaluation. These 890 document chunks were embedded by the fine-tuned and non–fine-tuned sentence transformer model, and the embeddings were stored in separate databases.

##### Patient-Centered Setup

The validated dataset from the cross-patient evaluation was used for the patient-centered RAG evaluation. As with the cross-patient setup, all documents included in this evaluation were sourced exclusively from patients who were held out from the training dataset. In total, the dataset consisted of 487 documents from 1 patient each and was divided into 2123 chunks. For each chunk, questions and corresponding answers were generated using the SauerkrautLM-SOLAR-Instruct model. As a result, 10,615 question-answer pairs were generated.

These additional questions and answers with the corresponding document chunks were annotated by 1 physician and 2 medical students. The students represented a range of academic backgrounds, including one student who has passed the first state examination for medicine and is currently in her seventh semester, and another who has studied and completed molecular biology and is currently in her second semester of pharmacy studies. Similar to the cross-patient data filtering described in the previous section, the annotators were instructed to remove any nonmedical questions and to include only those questions that would be asked in a realistic clinical setting. To gain insight into the quality of the annotations by measuring the agreement between the annotators, the annotators were divided into 2 groups. The first group consisted of 2 physicians, including 1 physician who had already annotated the cross-patient dataset and whose previously annotated data were reused for this comparison. The 2 medical students formed the second group. Each group received the same questions and answers. Interannotator agreement (IAA) was calculated based on precision, recall, and *F*_1_-score between the 2 annotators of 1 group. One annotator was designated as the ground truth, while the other served as the estimator. The annotated questions and answers were used as ground truth for patient-centered RAG evaluation. For each question, a filter was introduced, ensuring that only document chunks from a single patient were retrieved.

## Results

### Training Data Quality Assessment

To evaluate the reliability of the synthetically generated training corpus, a manual audit was conducted on a statistically random sample of 200 question-answer pairs, including corresponding document chunks, from the training dataset. This audit was performed by a physician to quantify the prevalence of generated artifacts. The analysis revealed that 36 pairs (18.0%) contained hallucinations, defined as questions for which the answer could not be derived from the provided document chunk. Additionally, 12 (6.0%) pairs contained factual errors or medically invalid reasoning. Consequently, 76.0% of the sample was verified as semantically accurate and clinically correct.

### IR Evaluation

**Figure 3.**
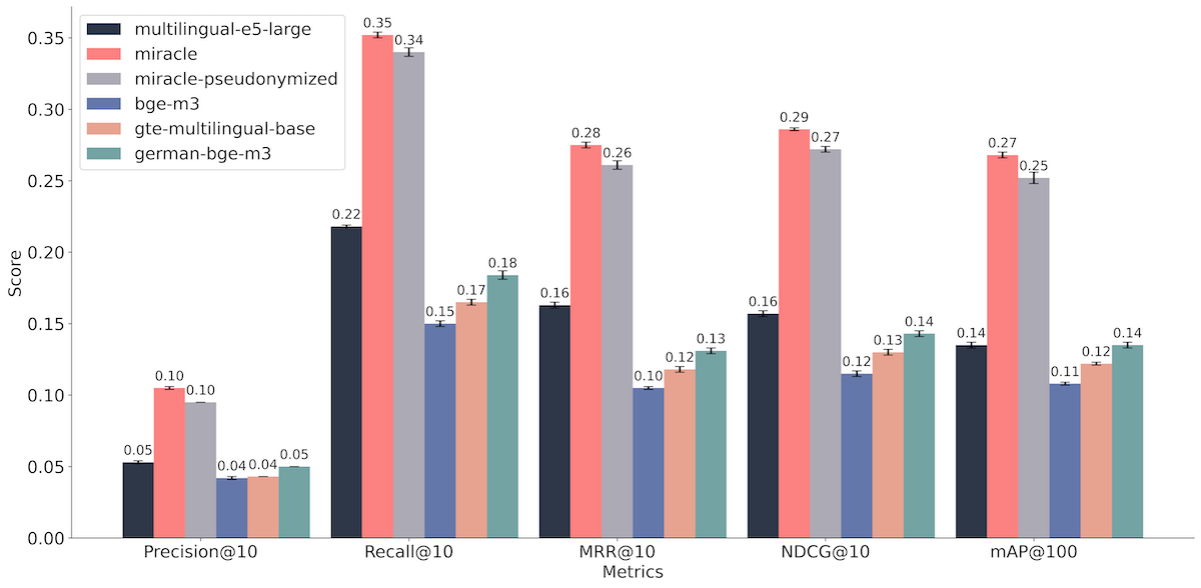
Comparative illustration of the information retrieval evaluation results for the German dataset. The results are presented for a k value of 10 for MRR, recall, precision, and NDCG. Abbreviations: mAP: mean average precision; MRR: mean reciprocal rank; NDCG: normalized discounted cumulative gain.

The IR evaluation metrics indicated a stronger performance of the fine-tuned embedding model, with a mAP@100 score improved from 0.14 to 0.27. The model fine-tuned on pseudonymized data also improved after training, achieving a mAP@100 of 0.25. The NDCG@10 metric increased after fine-tuning from 0.16 to 0.29 for the *miracle* model and to 0.27 for the pseudonymized model. Besides the fine-tuned models, the best-performing model for the German dataset was *multilingual-e5-large*. The *bge-m3* model scored a mAP@100 of 0.11 and a NDCG@10 of 0.12. The *gte-multilingual-base* model achieved slightly higher scores with a mAP@100 of 0.12 and a NDCG@10 of 0.13. The *german-bge-m3* model showed higher performances than the base *bge-m3* model, indicating that fine-tuning on German datasets improved language-specific capabilities. On the translated dataset, the *miracle* model also achieved the highest scores with improvements for mAP@100 from 0.12 to 0.27 and 0.25 for the pseudonymized model. The *bge-m3* model showed lower scores with a mAP@100 of 0.11. The *gte-multilingual-base* model scored a mAP@100 of 0.12. The results on the German dataset are visualized in Figure 3. The complete results for different k values and languages are appended in MultimediaAppendices 7  8.

### RAG Evaluation

#### Cross-Patient Results

The overall answer quality of the RAG system improved after fine-tuning the sentence transformer model. The BERTScore-F1 went up from 0.76 to 0.77. Improvements were also observed in both the ROUGE-2 score, which increased from 0.18 to 0.30, and the BLEURT score, which rose from 0.53 to 0.56. Other models such as *bge-m3* and *gte-multilingual-base* achieved lower scores in total, with a BERTScore-F1 score of 0.74 and a ROUGE-2 score of 0.17 for *bge-m3* and 0.16 for *gte-multilingual-base*. The fine-tuned variant of *bge-m3* (*german-bge-m3*) again showed better scores than the base model with a BERTScore-F1 of 0.75 and a ROUGE-2 score of 0.18. The highest scores for Contextual Precision and Contextual Recall were achieved by the pseudonymized model (0.82 and 0.89).

The results are in line with the answers generated by Qwen-3 (Multimedia Appendix 9). Because of the higher capabilities of this model, scores that are dependent on the generative part, such as BERTScore or ROUGE, are higher for every evaluated model. However, relative differences between the models stay intact, with the fine-tuned *miracle* model reaching the highest scores for most metrics (BERTScore F1: 0.78, ROUGE-2: 0.26) and showing improvements compared with the *multilingual-e5-large* (BERTScore F1: 0.76, ROUGE-2: 0.23).

For the translated dataset, the pseudonymized model achieved the highest scores with a BERTScore-F1 of 0.75 and a BLEURT score of 0.50. Of the non–fine-tuned models, the *bge-m3* model achieved the best scores for the English dataset with a BERTScore-F1 of 0.75 and a BLEURT score of 0.48. The RAG evaluation results for the German dataset are shown in Table 2. The results for the English dataset are provided in Multimedia Appendix 10.

**Table 2.** Retrieval Augmented Generation reevaluation results for the German dataset^a^.

Metric	multilingual-e5-large	miracle	miracle pseudonymized	bge-m3	gte-multilingual-base	german-bge-m3
BERTScore P^b^	0.733	*0.747* ^d^	0.736	0.725	0.725	0.728
BERTScore R^c^	0.783	*0.796* ^d^	0.792	0.760	0.756	0.779
BERTScore F1	0.756	*0.769* ^d^	0.762	0.742	0.739	0.747
BLEURT	0.533	*0.563* ^d^	0.551	0.542	0.539	0.553
ROUGE-1	0.327	*0.351* ^d^	0.342	0.319	0.311	0.327
ROUGE-2	0.179	*0.197* ^d^	0.194	0.167	0.159	0.175
ROUGE-L	0.276	*0.297* ^d^	0.292	0.269	0.258	0.274
Contextual P	0.685	0.779	*0.823* ^d^	0.626	0.567	0.676
Contextual R	0.774	0.860	*0.892* ^d^	0.726	0.676	0.783
Contextual Relevancy	0.307	*0.312* ^d^	0.309	0.274	0.268	0.310

a For the Retrieval Augmented Generation (RAG) evaluation, generated answers by the RAG system are compared with ground truth answers that were created by a large language model and filtered by a medical doctor. The higher the scores are, the more similar the answers are considered to the ground truth. The answers were generated by the *SauerkrautLM-Solar-Instruct* model.

b P: precision.

c R: recall.

d The values in italics highlight the highest scores in the respective category.

#### Patient-Centered Results

For the patient-centered RAG evaluation, the LLM-generated questions and answers (pairs) were filtered by 2 annotation groups. In Table 3, the results of the filtering process are displayed.

**Table 3.** Human filtering results of Retrieval Augmented Generation evaluation dataset^a^.

Annotator	Annotator group	Education	Pairs checked	Pairs filtered out, n (%)	IAA^b^
Annotator 1	Group 1	Physician	5556	2020 (36.36)	Precision=0.854; recall=0.963; *F*_1_-score=0.905
Annotator 2	Group 1	Physician	7081	1909 (26.96)	
Annotator 3	Group 2	Student (2nd semester)	3556	1813 (50.98)	Precision=0.763; recall=0.796; *F*_1_-score=0.779
Annotator 4	Group 2	Student (7th semester)	3839	1844 (48.03)	

a The Retrieval Augmented Generation evaluation dataset, which was partly created by a large language model, was given to 2 physicians and 2 medical students with the instruction to filter out questions or answer pairs that were medically unrelated. The dataset was divided into 2 parts, and each part was provided to 1 annotator group, resulting in 2 annotator groups. In the table, results of the human annotator process are displayed. The agreement between the annotators was calculated based on the overlapping annotated pairs of each annotator group (5556 for annotator group 1 and 3556 for annotator group 2).

b IAA: interannotator agreement.

Comparisons between annotator groups 1 and 2 show that there are differences in the number and percentage of filtered out question and answer pairs. While annotators 1 and 2 filtered out 36.36% and 26.96% of the dataset, annotators 3 and 4 filtered out 50.98% and 48.03%. IAA for group 1 was higher with an *F*_1_-score of 0.905 than for group 2, who reached an *F*_1_-score of 0.779.

The annotated question and answer pairs were used for the patient-centric RAG evaluation. For this evaluation scenario, the fine-tuned models again showed higher scores than the multilingual-e5-large, bge-m3, *german-bge-m3*, and gte-multilingual-base models. Compared with the results of the RAG evaluation without patient context, the differences between models have become smaller. The best-performing model, the model fine-tuned on pseudonymized data, achieved a BERTScore-F1 of 0.78, a BLEURT score of 0.61, and a ROUGE-1 score of 0.42. In comparison, the multilingual-e5-large reached a BERTScore-F1 of 0.78, a BLEURT score of 0.61, and a ROUGE-1 score of 0.41. Similar to the cross-patient evaluation, the highest scores for Contextual Precision and Contextual Recall were reached by the model trained on pseudonymized data. Interestingly, the highest score for Contextual Relevancy was achieved by the *german-bge-m3* model. The evaluation performed using the Qwen-3 as the generator showed similar results (Multimedia Appendix 11), with improvements for the fine-tuned models in all metrics compared with the *multilingual-e5-large* model. In Table 4, the RAG evaluation results for the patient-centric context are presented.

**Table 4.** Retrieval Augmented Generation patient-context evaluation results^a^.

Metric	multilingual-e5-large	miracle	miracle pseudonymized	bge-m3	gte-multilingual-base	german-bge-m3
BERTScore P^b^	0.763	*0.766* ^c^	*0.766* ^c^	0.764	0.761	0.756
BERTScore R^d^	0.795	*0.799* ^c^	*0.799* ^c^	0.796	0.793	0.793
BERTScore F1	0.778	*0.781* ^c^	*0.781* ^c^	0.778	0.775	0.773
BLEURT	0.608	*0.611* ^c^	*0.611* ^c^	0.608	0.604	0.593
ROUGE-1	0.414	0.420	*0.421* ^c^	0.415	0.407	0.394
ROUGE-2	0.249	*0.256* ^c^	*0.256* ^c^	0.250	0.242	0.232
ROUGE-L	0.355	0.361	*0.362* ^c^	0.355	0.349	0.335
Contextual P	0.868	0.918	*0.927* ^c^	0.858	0.824	0.880
Contextual R	0.927	0.938	*0.939* ^c^	0.925	0.909	0.929
Contextual Relevancy	0.239	0.241	0.241	0.235	0.233	*0.242* ^c^

a For the Retrieval Augmented Generation (RAG) evaluation, generated answers by the RAG system are compared with ground truth answers that were created by a large language model and filtered by 4 human annotators. The higher the scores are, the more similar the answers are considered to the ground truth. The answers were generated by the *SauerkrautLM-Solar-Instruct* model.

b P: precision.

c The values in italics highlight the highest scores in the respective category.

d R: recall.

For more nuanced performance differences between the models, the patient-centric performances were broken down by document category (Multimedia Appendix 12). For discharge letters, radiology reports, and operation notes, the fine-tuned models show the highest scores, with only slight differences between the models. The results on pathology reports indicate that there is a stronger difference in performance between models, with the *gte-multilingual-base* achieving the highest BERTScore-F1 and ROUGE scores.

## Discussion

### Principal Findings

This study developed and tested language-specific clinical embedding models specifically trained on verified hospital documents for medical RAG applications and demonstrated their higher accuracy metrics. The introduced approach and models address an important gap in NLP applications for health care by improving clinical language comprehension for multiple languages and by optimizing RAG systems within medical information environments. Without domain fine-tuning, it is challenging for embedding models to comprehend clinical language and contexts, as they are not trained on real-world medical documents. The integration of these models as retrieval components in RAG systems may lead to undesired or medically irrelevant results. Fine-tuning embedding models on medical real-world data represent an advancement in the field of medical NLP and improve artificial intelligence–driven health care by facilitating the understanding of clinical language.

Previous research focused on English language models for NLP and has proven its application in various clinical settings [38,39]. This strategy, however, has faced shortcomings in performance, in particular, in the multilanguage setting. Our models have been fine-tuned on German medical data, a language not frequently used in medical NLP [20], and whose documents are currently less abundant. The presented work not only complements but also diversifies the linguistic scope of medical NLP research and its clinical applicability. The extracted dataset from 4 medical application areas had highly nuanced and domain-specific medical language. By using an LLM to generate question and answer pairs for these documents, an extensive dataset with approximately 11 million pairs served as a foundation for fine-tuning and evaluation. Different evaluation benchmarks introduced in this study provide insights into improvements achieved after fine-tuning the models on this dataset.

Importantly, these fine-tuned context and language-sensitive miracle models surpassed the state-of-the-art *multilingual-e5-large*, *bge-m3*, *german-bge-m3,* and *gte-multilingual-base* models in 2 evaluation scenarios. The model trained on real-world data showed the best overall results for the German evaluation datasets. More interestingly, the model fine-tuned on pseudonymized data achieved similar results on the IR and even better results for the RAG evaluation on the English-translated dataset.

One hypothesis is that the pseudonymization process may have resulted in an improved machine readability of certain phrases, which in turn may have positively influenced the quality of the translation. However, the models fine-tuned on translated data overall showed lower scores than the models trained on real-world data. Noise and possibly difficult translations of abbreviations might have influenced the dataset quality.

Performance comparisons, particularly with the *german-bge-m3* model, showed that language-specific fine-tuning generally can improve retrieval scores. However, the improvements achieved by fine-tuning on real-world clinical data indicate that further enhancements can be achieved by domain adaptation.

The results of the patient-centered RAG evaluation show that the fine-tuned models are able to find suitable text passages more accurately in a patient question answering setting. The category-wise results indicate differences in performance depending on the document type. As documents from different clinics and departments are written in different styles with varying language and form, the retrieval of suitable text passages is highly dependent on the ability of the models to semantically understand and interpret medical language. The highest scores were achieved for discharge letters and radiology reports, indicating simpler language compared with pathology reports and surgical reports, which may contain more specific and nuanced language.

By having physicians and medical students annotate the evaluation dataset for the patient-centered evaluation, cautious estimates can be made about the quality of the dataset. The human annotation results highlight both the limitations and effectiveness of synthetic data generation in medical contexts. While 27%‐51% of LLM-generated pairs required filtering, the remaining validated content still provided sufficient evaluation data. The variation in filtering rates between annotators likely reflects differences in clinical experience and criteria for data quality. Despite these quality variations, the consistent performance improvements observed across all evaluation scenarios suggest that the signal-to-noise ratio in the synthetic training data was sufficient for effective model fine-tuning. These findings indicate that while expert validation improves data quality, synthetic generation can still yield functional training corpora for specialized domains such as clinical NLP.

Serving as the foundation for our research, the provided dataset consisting of real-world clinical texts and LLM-generated question-answer pairs offers a comprehensive training resource that captures the nuances of medical terminology and clinical reasoning. Building upon this dataset, the presented technologies indicate a potential to transform how health care professionals access and use medical information. An example of how this technology could be integrated into clinical routine is visualized in Multimedia Appendix 13.

Regarding operational implementation in hospital IT infrastructures, the deployment of the fine-tuned embedding model presents a low barrier to entry. Unlike the training phase or the generative components of RAG systems, which often require GPU acceleration, the retrieval component based on dense vector embeddings is computationally efficient during inference. The model can be deployed on standard, CPU-based server environments commonly found in hospital data centers. This efficiency allows for seamless integration into existing clinical workflows and decision support systems without imposing additional infrastructure costs or latency, making the solution highly scalable for real-time IR across large patient cohorts.

### Limitations

While our models indicate a step forward for the development of clinical embedding models for RAG systems, they are not without limitations. The dataset, though comprehensive, is limited to 4 types of clinical documents and may benefit from expansion by including a greater variety of medical texts. Furthermore, as the documents were extracted from 1 site only, the model is susceptible to template overfitting. Clinical documents from a single institution often share rigid structural templates and specific formatting cues, creating a risk that the model learns to associate relevance with these institutional artifacts rather than purely semantic content. Consequently, the performance observed here may not fully transfer to institutions with different documentation standards without local fine-tuning. Additionally, the English language model was trained on machine-translated data using the WMT’19 German-to-English translation model, with translation quality validated on only 30 physician-reviewed samples. This sample size is statistically insufficient to ensure translation accuracy across the entire corpus of 400,000 clinical documents. Machine translation of specialized medical terminology, abbreviations, and context-dependent clinical language may introduce systematic translation artifacts that differ from native English clinical documentation. The models fine-tuned on translated data should therefore be considered a proof of concept for cross-lingual transfer learning rather than a validated clinical tool ready for deployment in English-speaking health care settings.

Furthermore, the current evaluation compared performance exclusively against multilingual baselines to align with the model’s architectural foundation. We did not benchmark against embedding models trained exclusively on English clinical data. Consequently, while our results demonstrate the model’s efficacy within a multilingual framework, its relative performance against specialized, monolingual English clinical models remains to be validated in future studies.

A circular evaluation bias may be inherent to the experimental design, as the test questions were synthetically generated using the same LLM family used for training. This shared origin implies that the evaluation might partially measure the model’s ability to recognize synthetic linguistic patterns rather than organic human queries. To mitigate this, the Qwen3-235B-A22B-Instruct-2507-FP8 was additionally used as the generator part of the RAG system. The results showed overall higher scores but no significant relative performance differences between the evaluated embedding models.

Furthermore, the IR evaluation was conducted in a cross-patient setting where synthetically generated questions could match document chunks from multiple patients, with all matches treated as relevant. A limitation of this methodology is that it relies on identifying duplicate questions, which inherently introduces a selection bias toward generic queries (eg, “What is the diagnosis?”) that are likely to appear multiple times across the corpus. Consequently, complex, specific, or nuanced questions that are unique to a single patient case were systematically excluded. This bias may result in an overestimation of performance on simple lookup tasks while underrepresenting the challenges of complex clinical reasoning. Additionally, treating all matches as relevant introduces potential label noise, as generic questions may retrieve contextually appropriate chunks from different patients that answer different clinical questions. However, this evaluation design does capture legitimate clinical use cases, including searching for similar patient presentations, researching treatment outcomes across cases, or conducting retrospective cohort analyses. To address patient-specific scenarios, we conducted a separate patient-centered RAG evaluation with retrieval restricted to single patients, which demonstrated the models’ effectiveness in realistic clinical contexts.

The observed low absolute IR performance metrics must be interpreted in the context of the extensive search space, which comprised 173,933 document chunks across 26,358 patients. This high-dimensional setting renders specific retrieval statistically challenging compared with constrained, single-patient scenarios. The evaluation methodology relied on identifying exact duplicate synthetic questions to define ground truth. This rigid definition of relevance likely penalized semantically redundant chunks that contained relevant information but generated slightly different synthetic questions. Such instances are treated as irrelevant in our evaluation, resulting in potentially high false-negative rates. Consequently, strict metrics such as Accuracy@1 may underestimate the model’s true clinical usefulness, as they measure the model’s ability to retrieve the exact source chunk rather than its ability to retrieve any chunk containing the correct semantic answer. The RAG evaluation does not directly measure the IR but the answer quality, which also could be biased by the LLM component of the RAG system. However, the combination of different evaluation scenarios allows for a more balanced and diverse look at the performance of the models.

In the patient-centered RAG evaluation, the fine-tuned miracle model consistently achieved the highest numerical scores across all metrics. However, we explicitly acknowledge that the performance differences compared with the multilingual-e5-large baseline were marginal. A formal statistical significance test was not performed for these specific evaluation metrics. Consequently, the statistical significance of these performance differences has not been established. It remains possible that the observed marginal improvements fall within the margin of error and may result from random variance rather than a systematic model advantage. This stands in contrast to the larger performance deltas observed in the cross-patient settings. We hypothesize that this convergence may be attributable to a potential saturation effect caused by the constrained search space. In the patient-centered setting, the retrieval pool is restricted to a single patient’s history (typically comprising fewer than 30 document chunks). With such a limited number of distractor chunks, the baseline model already achieves near-ceiling retrieval performance, potentially masking the benefits of domain-specific fine-tuning that become more apparent in larger, high-dimensional retrieval spaces.

We further acknowledge a discrepancy in the IAA observed during the dataset validation. While the physician group achieved a high *F*_1_-score of 0.905, the medical student group achieved a lower score of 0.779. This variance is potentially attributable to differences in clinical experience, as students may lack the consolidated clinical intuition required to consistently apply exclusion criteria to borderline cases. As the student-annotated portion of the dataset was used as ground truth without a secondary confirmation review by a senior clinician, this introduces a potential for label noise. Consequently, the evaluation metrics for the patient-centered scenario should be interpreted with this underlying annotator variability in mind.

A notable finding in our RAG evaluation was the consistently low Contextual Relevancy scores (approximately 0.24) observed across all models. This indicates that roughly three-quarters of the content within a retrieved chunk was classified as nonrelevant to the specific query by the judge model. We attribute this to the high information density characteristic of clinical documentation. Even with a constrained text segment of 450 characters, clinical notes frequently contain multiple distinct, packed statements. Consequently, while the chunk contains the correct answer, it inevitably includes competing clinical facts that lower the relevancy ratio. This highlights a persistent challenge in medical IR: finding an optimal granularity that isolates specific findings without destroying the narrative context.

A further limitation concerns the text segmentation strategy. We used a fixed chunk size of 450 characters with an 80-character overlap. While this granularity enhances the precision of retrieval for specific clinical facts, it risks context fragmentation, particularly for complex temporal reasoning or negation scopes that span across multiple segments (eg, a diagnosis mentioned at the beginning of a letter and a treatment plan at the end). Furthermore, an alternative atomic-level chunking strategy (eg, sentence-level splitting) could theoretically maximize relevancy scores for closed-ended questions (eg, “What is the patient’s sodium level?”). However, we deliberately avoided this approach to prioritize semantic coherence. Atomic splitting risks fragmenting the narrative flow essential for open-ended clinical reasoning (eg, linking a diagnosis to its ruling out criteria in the next sentence). Therefore, the reported low relevancy scores reflect a trade-off where we accepted lower precision in signal-to-noise ratio to preserve the integrity of the local clinical context.

LLMs, including the *SauerkrautLM-SOLAR-Instruct* model used for data generation, are susceptible to hallucinations and may generate factually incorrect or clinically inappropriate content [40]. This introduces potential noise into the training dataset that could propagate to the fine-tuned embedding models. Analysis of a random sample indicated that approximately 24% of the generated question-answer pairs contained hallucinations or factual inaccuracies. This introduces a safety concern, as this specific type of noise has the potential to propagate into the embedding space, effectively teaching the model incorrect semantic associations. While our downstream performance suggests that the valid signal was dominant, we explicitly warn that the model was trained on a corpus with a known error rate. Therefore, the resulting embeddings should not be treated as ground truth. Any application of these models in clinical practice must mandate robust downstream filtering and expert human verification to identify and intercept potential retrieval errors stemming from these training artifacts. For evaluation, the question-answer pairs were also initially generated by an LLM. However, a rigorous human-in-the-loop validation process involving physicians and medical students was conducted to mitigate the risks associated with purely synthetic benchmarks. This resulted in a filtering rate of approximately 27%-51%, ensuring that the final test set represents a human-verified standard of clinical relevance.

A further limitation concerns the evaluation scope regarding negative testing. While the RAG system prompt included instructions to refuse answering if the context was insufficient (eg, stating “’I don’t know”), our quantitative evaluation focused exclusively on positive retrieval scenarios, specifically cases where the answer was present in the ground truth. We did not systematically assess the model’s ability to correctly reject irrelevant context or quantify the rate of hallucinations when no suitable information is retrieved. Validating this refusal capability is an important safety requirement for clinical deployment. Without it, there is a risk that the system might confidently hallucinate answers based on retrieved but irrelevant distractor chunks.

Relatedly, the quantitative assessment of the RAG system relied on automated semantic similarity metrics such as BERTScore, BLEURT, and ROUGE. While these metrics are standard benchmarks for measuring the linguistic overlap between generated answers and ground truth, they are not designed to verify factual medical precision. High semantic scores can occasionally mask critical factual errors, such as laterality swaps (eg, “left” vs “right”) or negation flips (eg, “no evidence of” vs “evidence of”), which may have high lexical overlap with the correct answer.

### Future Work

Future validation on external datasets from different sites and the inclusion of additional document categories would strengthen the approach and indicate broader applicability. To facilitate this necessary next step, we have publicly released the models fine-tuned on pseudonymized and translated data. This allows the broader research community to validate the miracle models on their local datasets, thereby establishing their generalizability across different clinical environments in future work.

While in the study, quantitative metrics were assessed for comparison of embedding models, evaluation with clinicians is an important next step to evaluate clinical performance for real-world scenarios. Evaluation with end users could give insights into the integration of the fine-tuned models in a real-world setting and should be investigated in future studies. Future clinical validation studies must use expert human verification to audit generated responses for specific types of high-risk factual hallucinations. Future studies should also include dedicated patient-specific IR benchmarks with queries explicitly designed for individual patient contexts. Additionally, it remains to be validated whether the domain-specific fine-tuning makes the model more robust in extracting correct answers or potentially more prone to hallucinating responses due to an increased bias toward using retrieved clinical text.

Comparisons between the fine-tuned models and domain-adapted embedding models could strengthen the approach and give insights into the quality of the QA dataset. However, to our knowledge, there are no publicly available fine-tuned sentence transformer models trained on German medical real-world data.

The retrieval parameter k (number of retrieved chunks) was fixed at 3 for all RAG experiments. We acknowledge that this parameter was not empirically optimized through ablation studies within this work. While the parameter value was selected to provide a reasonable balance between context sufficiency and noise reduction, it is possible that the choice of k influenced the reported performance metrics. Determining the optimal retrieval depth or using dynamic threshold-based retrieval strategies requires further investigation.

Although the recursive splitting method respects paragraph boundaries to maintain local semantic integrity, future studies should explore adaptive segmentation strategies. While section-aware chunking or hierarchical retrieval can better capture long-range dependencies, future research should also investigate hybrid segmentation pipelines. Such approaches could dynamically use atomic-level chunking for high-density, structured data (eg, laboratory values and medication lists) to maximize precision, while retaining larger, section-aware windows for unstructured narrative prose (eg, anamnesis and discharge summary) to preserve semantic flow. Investigating the optimal combination of these strategies remains an important step toward production-grade clinical RAG systems.

Furthermore, while this study evaluated performance using dense vector retrieval, clinical decision-making often requires reasoning across complex, interrelated entities (eg, drug-drug interactions or temporal disease progressions). Future research should explore integrating these fine-tuned embedding models into Graph Retrieval Augmented Generation systems. In such architectures, our domain-adapted embeddings could serve to enhance node resolution and relationship traversal within clinical knowledge graphs, potentially addressing the limitations of purely vector-based retrieval for multihop reasoning tasks.

Finally, our evaluation focused specifically on the performance of dense vector retrieval. We did not benchmark against lexical approaches such as BM25 or multistage pipelines involving cross-encoder reranking or multivector architectures such as ColBERT. While lexical search can be effective for exact keyword matching, it often fails to capture the semantic nuances of medical terminology. However, we acknowledge that in a production-grade clinical RAG system, the fine-tuned miracle embeddings presented here would likely serve as the retrieval foundation within a hybrid pipeline, potentially supplemented by keyword search and followed by a reranking step to maximize precision. Further research should also explore section-aware chunking or hierarchical retrieval approaches to better capture long-range dependencies in clinical narratives.

### Conclusions

This study demonstrates that embedding models fine-tuned on real-world clinical documents can outperform existing state-of-the-art multilingual models for medical IR and RAG applications. The approach of generating synthetic question-answer pairs from clinical documents using LLMs provides a scalable methodology for creating domain-specific training data while maintaining patient privacy through pseudonymization. The models’ effectiveness across both German and English datasets indicates applicability beyond single-language contexts. This work establishes a reproducible framework for developing specialized embedding models that address the gap between general-purpose language models and the specific requirements of health care documentation, particularly for non-English medical settings where such resources are limited.

## Supplementary material

10.2196/82997 Multimedia Appendix 1 Additional information about cosine similarity.

10.2196/82997 Multimedia Appendix 2 Additional information about precision, recall, and *F*_1_-score.

10.2196/82997 Multimedia Appendix 3 Parameter size of evaluated models.

10.2196/82997 Multimedia Appendix 4 Additional information about information retrieval metrics.

10.2196/82997 Multimedia Appendix 5 Additional detailed information about Retrieval Augmented Generation metrics.

10.2196/82997 Multimedia Appendix 6 Prompt example for evaluation of Retrieval Augmented Generation systems.

10.2196/82997 Multimedia Appendix 7 Information retrieval evaluation results on the German dataset.

10.2196/82997 Multimedia Appendix 8 Information retrieval evaluation results on English dataset.

10.2196/82997 Multimedia Appendix 9 Retrieval Augmented Generation evaluation results on the German dataset in a cross-patient setting with answers generated by Qwen-3.

10.2196/82997 Multimedia Appendix 10 Retrieval Augmented Generation evaluation results on English dataset in cross-patient setting.

10.2196/82997 Multimedia Appendix 11 Retrieval Augmented Generation evaluation results on the German dataset in a patient-centered setting with answers generated by Qwen-3.

10.2196/82997 Multimedia Appendix 12 Retrieval Augmented Generation patient-centered evaluation results per document category.

10.2196/82997 Multimedia Appendix 13 Mock-up implementation of clinical Retrieval Augmented Generation system with integration into electronic health record for patient question answering.

## Abbreviations

IAA: interannotator agreement
IR: information retrieval
LLM: large language model
MAP: mean average precision
NDCG: normalized discounted cumulative gain
NLP: natural language processing
RAG: Retrieval Augmented Generation
