# Supplementary material for: Improving Retrieval Augmented Generation for Health Care by Fine-Tuning Clinical Embedding Models: Development and Evaluation Study
Source: J Med Internet Res. 2026 Mar 25;28:e82997. doi: 10.2196/82997 (PMC13016438; doi:10.2196/82997)
Supplement: Multimedia Appendix 1 [file jmir-v28-e82997-s001.docx]

# Multimedia Appendix 1

## Additional Information about Cosine Similarity.

With the help of cosine similarity, the similarity between two vectors can be measured. In this evaluation setup, the two vectors are a query and a passage. For two vectors $x$ and $y$, the cosine similarity function is defined as $sim(x,y) =\frac{x \cdot y}{\left| \left| x \right| \right| \left| \left| y \right| \right|}$, where $\left| \left| x \right| \right|$ is the Euclidean norm of vector $x = (x_{1}, x_{2},...,x_{p})$ defined as $\sqrt{{x_{1}}^{2}+{x_{2}}^{2}+ ...+{x_{p}}^{2}}$. In the same way, $\left| \left| y \right| \right|$ is defined as the norm Euclidean norm of vector $y$. In other words, the angle between two vectors is computed. A cosine value of 0 means the two vectors are orthogonal to each other and have no match. A cosine value close to 1 indicates a smaller angle and a greater degree of alignment between the vectors.
