# Supplementary material for: Improving Retrieval Augmented Generation for Health Care by Fine-Tuning Clinical Embedding Models: Development and Evaluation Study
Source: J Med Internet Res. 2026 Mar 25;28:e82997. doi: 10.2196/82997 (PMC13016438; doi:10.2196/82997)
Supplement: Multimedia Appendix 2 [file jmir-v28-e82997-s002.docx]

# Multimedia Appendix 2

## Additional Information about Precision, Recall and F1-score.

To evaluate the performance for this evaluation task, precision (P), recall (R), and F1-score, defined as $P=TP/(TP+FP)$, $R=TP/(TP+FN)$, and $F1=2\times precision\times recall/(precision+recall)$, were calculated. Precision is calculated as the ratio of true positives (TP) to the sum of TP and false positives (FP). In other words, the proportion of TP predictions among all positive predictions is measured. Recall is calculated as the ratio of TP to the sum of TP and false negatives (FN) and measures the proportion of TP predictions among all actual positive instances. The F1-score harmoniously combines precision and recall, providing a balanced assessment of model performance.
