# Supplementary material for: Improving Retrieval Augmented Generation for Health Care by Fine-Tuning Clinical Embedding Models: Development and Evaluation Study
Source: J Med Internet Res. 2026 Mar 25;28:e82997. doi: 10.2196/82997 (PMC13016438; doi:10.2196/82997)
Supplement: Multimedia Appendix 3 [file jmir-v28-e82997-s003.docx]

# Multimedia Appendix 3

## Parameter Size of Evaluated Models.

| **Model** | **Parameter Size** | **Model Card** |
| --- | --- | --- |
| multilingual-e5-large | 600M | <https://huggingface.co/intfloat/multilingual-e5-large> |
| miracle | 600M | <https://huggingface.co/ikim-uk-essen/miracle> |
| bge-m3 | 600M | <https://huggingface.co/BAAI/bge-m3> |
| gte-multilingual-base | 300M | <https://huggingface.co/Alibaba-NLP/gte-multilingual-base> |
| German-RAG-bge-m3 | 600M | <https://huggingface.co/avemio/German-RAG-BGE-M3-MERGED-x-SNOWFLAKE-ARCTIC-HESSIAN-AI> |
