# Supplementary material for: Improving Retrieval Augmented Generation for Health Care by Fine-Tuning Clinical Embedding Models: Development and Evaluation Study
Source: J Med Internet Res. 2026 Mar 25;28:e82997. doi: 10.2196/82997 (PMC13016438; doi:10.2196/82997)
Supplement: Multimedia Appendix 4 [file jmir-v28-e82997-s004.docx]

# Multimedia Appendix 4

## Additional Information about Information Retrieval Metrics.

*Precision@K* is defined as $Precision@K = \frac{true positives@k}{(true positives@k) + (false positives@k)}$ and quantifies how many of the elements of the top-K results were relevant. For example if only one element in the top 2 results were relevant for a K value of 2, the *Precision@2* would be 0.5. *Recall@K* is defined as $Recall@K = \frac{true positives@k}{(true positives@k) + (false negatives@k)}$ and expresses the number of actual relevant results out of all actual relevant results for the query. For a k value of 2, if only one item was considered to be relevant, but three items were actually relevant, the *Recall@2* would be approximately 0.33. *Accuracy@K* is defined as $Accuracy@K = \frac{1}{N} \sum_{i=1}^{N} I\left( y_{i}\in\hat{y}_{i} \right)$ where $N$ is the total number of samples in the dataset, $I(\cdot)$ is the indicator function, $y_{i}$ is the actual ground-truth label for the $i$-th sample, and $\hat{y}_{i}$ is the set of the K classes with the highest predicted probabilities for the $i$-th sample. The indicator function equals 1 if the condition inside the parenthesis is true, and 0 otherwise. In short, Top K accuracy is calculated as the fraction of samples for which the true label is among the model's top K most probable predicted labels.

Another important evaluation benchmark in information retrieval is *Mean Reciprocal Rank* (*MRR*). In contrast to *Precision@K* or *Recall@K*, the rank of the relevant result is also taken into account for this metric. It is defined as $MRR =\frac{1}{\left| Q \right|}\sum_{i=1}^{\left| Q \right|} \frac{1}{{rank}_{i}}$ where $\left| Q \right|$ denotes the total number of queries and ${rank}_{i}$the rank of the first relevant result. Thus only the first relevant result is used for the calculation of the metric, all other relevant results are ignored.

For *average precision* on the other hand all of the relevant results are considered for the calculation. It is defined as $AP = \frac{\sum_{k=1}^{n} (P(k)*rel(k))}{number of relevant items}$ where $rel(k)$ is an indicator that takes a value of 1 if the element at rank k is relevant. $P(k)$ is the *Precision@K* metric, so it essentially can be calculated by using this metric at different k values and dividing it by the total number of relevant items. To calculate the average precision over $N$ queries, the Mean average precision is defined as $mAP = \frac{1}{N}\sum_{i=1}^{N} {AP}_{i}$ and is simply the mean of AP over the number of queries $N$.

The relevance of a user query can be calculated with discounted cumulative gain (DCG). This metric also takes into account the order of the retrieved items and gives less weight to relevant items that appear lower in the list. It is defined as $DCG@K = \sum_{k=1}^{K} \frac{{rel}_{i}}{㏒_{2}(i +1)}$ where ${rel}_{i}$ is the relevance score of the item at position $i$. The introduction of a logarithmic penalty function results in a lower DCG for items that are relevant but appear further down the retrieved list in comparison to items that are relevant and appear at the top positions. The normalized DCG (NDCG) is defined as $NDCG@K = \frac{DCG@K}{IDCG@K}$ and divides the $DCG$ by the ideal ranking $IDCG$. IDCG represents the maximum achievable DCG with the same set of relevance scores, but in a ranking order that would be considered optimal. Normalized DCG allows for fair comparisons between lists of different lengths and relevance scores.
