# Supplementary material for: Improving Retrieval Augmented Generation for Health Care by Fine-Tuning Clinical Embedding Models: Development and Evaluation Study
Source: J Med Internet Res. 2026 Mar 25;28:e82997. doi: 10.2196/82997 (PMC13016438; doi:10.2196/82997)
Supplement: Multimedia Appendix 5 [file jmir-v28-e82997-s005.docx]

# Multimedia Appendix 5

## Additional Detailed Information about RAG metrics.

In order to assess the quality of the output produced by the LLM in comparison to the ground truth, a range of metrics commonly applied in the field of natural language processing are employed. *ROUGE* is a set of metrics that are used for the comparison of the quality of a generated text with a reference text. Lin, the creator of the ROUGE metrics, showed in her study that ROUGE scores correlate with human judgements. It was originally developed for machine translation tasks but is also employed to evaluate answers from language models with a ground truth. ROUGE-1 measures the overlap of single words (unigrams) and ROUGE-2 the overlap of pairs of adjacent words (bigrams) between the generated summary and the reference summary. To check the match of longer subsequences, the ROUGE-L metric can be used. A subsequence is a sequence of words in the same order, not necessarily consecutive. BLEU is another method of automatic machine translation evaluation that allows comparing a candidate text to one or more reference texts. Papineni et al.developed BLEU for automatic machine evaluation and showed in their study that it correlates with human judges.

Because BLEU and ROUGE metrics are only sensitive to lexical variation, they cannot appropriately reward semantic or syntactic variations of a given reference. For natural language generation tasks, semantic comparison between generated and reference text is necessary to evaluate the performance. Therefore in recent years, new metrics were developed that introduce learnable components. BLEURTis an evaluation metric based on BERT that can assess human judgments with a few thousand possibly biased examples. The model was pre-trained on millions of synthetic reference-candidate pairs and fine-tuned on task-specific ratings. BERTScoreis an alternative automatic evaluation metric that incorporates the semantic relationship between the generated text and the reference text. BERTScore employs the pre-trained contextual embeddings derived from BERT to identify similarities between words in candidate and reference sentences through cosine similarity. Its efficacy in aligning with human judgment on sentence-level and system-level evaluation has been demonstrated by Zhang et al. Furthermore, BERTScore facilitates the computation of Precision, Recall, and F1 measure, which can be instrumental in assessing the performance of diverse language generation tasks. Different models can be used for the computation of semantic similarities of BERTScore. Since the language of the underlying data for this study is German, the *bert-base-multilingual-cased* modelwas employed to calculate the cosine similarity between generated and reference texts.

Besides these metrics to compare the output with a ground truth, there are other metrics to measure the retrieval quality of RAG systems. Established metrics include Contextual Precision, Contextual Recall and Contextual Relevancy. All these metrics use an LLM-as-a-judge to measure the quality of the retrieval in the RAG-pipeline.

Contextual Precision is defined as $Contextual Precision = \frac{1}{Number of Relevant Nodes}\sum_{k=1}^{n} (\frac{number of relevant items}{k}\times r_{k})$, where *k* is the ${(i+1)}^{th}$ node in the retrieval context, *n* is the length of the retrieval context and $r_{k}$ is the binary relevance of the $k^{th}$ node in the retrieval context. A higher contextual precision indicates the ability of the retrieval model to correctly rank relevant nodes in the retrieved context. Contextual Recall is defined as $Contextual Recall= \frac{Number of Attributable Statements}{Total Number of Statements}$, where $Total Number of Statements$ denotes the generated output of the RAG-pipeline and $Number of Attributable Statements$ represents the number of retrieval nodes that are relevant for the generation of the output. A higher Contextual Recall represents a greater ability of the retrieval part of the RAG system to capture all relevant information from the total available retrieved sets. Contextual Relevance is defined as $Contextual Relevance= \frac{Number of Relevant Statements}{Total Number of Statements}$, where $Total Number of Statements$ stands for the statements made in each retrieved node and $Number of Relevant Statements$ specifies the number of actually relevant nodes for the user question.
