# Supplementary material for: Improving Retrieval Augmented Generation for Health Care by Fine-Tuning Clinical Embedding Models: Development and Evaluation Study
Source: J Med Internet Res. 2026 Mar 25;28:e82997. doi: 10.2196/82997 (PMC13016438; doi:10.2196/82997)
Supplement: Multimedia Appendix 6 [file jmir-v28-e82997-s006.docx]

# Multimedia Appendix 6

## Prompt Example for Evaluation of Retrieval Augmented Generation Systems.

The example shows the prompt, the question and the passages that were retrieved by the sentence transformer model and given as context to the LLM to answer the question. The LLM generated an answer that was compared to the ground truth answer using metrics like ROUGE, BLEURT or BERTScore.

| **Prompt:**  ### Task: Answer the following question using the context provided.  If the context doesn't contain the answer, just say that you don't know the answer, don't try to make up an answer.  ### Question: What is the assessment of the cervical spine (cervical-spine area) and have any fractures or unusual findings been determined in the cervical soft tissues?  ### Context:  *"passage: In the CTA actual representation of the supraaortic and intracranial vessels. No vessel occlusions. No stenosis. No proof of a vascular dissection. Cervical spinal canal: normal dorsal alignment. No evidence of fractures. As far as the CT does not detect an intraspinal or paravertebral hematoma. Normal spinal canal wideness. Unremarkable representation of cervical soft tissues."*  *"passage: No stenosis, no evidence of vascular dissection. Lateral spinal canal: Proper dorsal alignment. No proof of fractures. As far as CT can be assessed, no intraspinal or paravertebral hematoma. Normal width of the spinal canal. Inconspicuous depiction of cervical soft tissues. Result:"*  *"passage: Normal representation of the cervical spine and upper cervical spine without vertebral bodies that have reduced in height. Real alignment when lying down. No evidence of fracture. No intraspinal or paravertebral haematoma assessed by CT. Normal width of the spinal canal. Unobtrusive depiction of cervical soft tissues and the basally detected neurocranium."*    ### Generated Answer: From the provided context, the assessment of the cervical spine shows a normal alignment, no evidence of fractures, and no unusual findings in the cervical soft tissues. There is also no indication of intraspinal or paravertebral hematomas based on the CT scans.  ### Ground Truth Answer: The cervical spine evaluation shows a regular dorsal alignment, without fractures or an inconspicuous representation of the cervical soft tissues. |
| --- |
