# Supplementary material for: Improving Retrieval Augmented Generation for Health Care by Fine-Tuning Clinical Embedding Models: Development and Evaluation Study
Source: J Med Internet Res. 2026 Mar 25;28:e82997. doi: 10.2196/82997 (PMC13016438; doi:10.2196/82997)
Supplement: Multimedia Appendix 7 [file jmir-v28-e82997-s007.docx]

# Multimedia Appendix 7

## Information retrieval evaluation results on German dataset.

For the information retrieval evaluation, duplicate questions in the dataset were identified. This way, multiple document passages for each question could be provided which essentially is an information retrieval task. The evaluation was performed with different k-values and by measuring the distance between question and chunk pair using cosine similarity. MRR stands for Mean Reciprocal Rank, NDGC stands for Normalized Discounted Gain and mAP stands for Mean Average Precision.

| **Metric** | multilingual-e5-large | miracle | miracle-  pseudonym-ized | bge-m3 | gte-multilingual-base |
| --- | --- | --- | --- | --- | --- |
| Accuracy@1 | 0.103 | **0.203** | 0.192 | 0.074 | 0.086 |
| Precision@1 | 0.103 | **0.203** | 0.192 | 0.074 | 0.086 |
| Recall@1 | 0.046 | **0.083** | 0.082 | 0.031 | 0.038 |
| Accuracy@3 | 0.189 | **0.309** | 0.293 | 0.120 | 0.134 |
| Precision@3 | 0.087 | **0.184** | 0.173 | 0.071 | 0.092 |
| Recall@3 | 0.112 | **0.218** | 0.212 | 0.087 | 0.105 |
| Accuracy@5 | 0.246 | **0.370** | 0.351 | 0.147 | 0.160 |
| Precision@5 | 0.073 | **0.147** | 0.136 | 0.058 | 0.064 |
| Recall@5 | 0.153 | **0.273** | 0.264 | 0.112 | 0.132 |
| Accuracy@10 | 0.325 | **0.463** | 0.442 | 0.190 | 0.199 |
| Precision@10 | 0.053 | **0.105** | 0.095 | 0.042 | 0.043 |
| Recall@10 | 0.218 | **0.352** | 0.340 | 0.150 | 0.165 |
| MRR@10 | 0.163 | **0.275** | 0.261 | 0.105 | 0.118 |
| NDCG@10 | 0.157 | **0.286** | 0.272 | 0.115 | 0.130 |
| mAP@100 | 0.135 | **0.268** | 0.254 | 0.108 | 0.122 |
