# Supplementary material for: Improving Retrieval Augmented Generation for Health Care by Fine-Tuning Clinical Embedding Models: Development and Evaluation Study
Source: J Med Internet Res. 2026 Mar 25;28:e82997. doi: 10.2196/82997 (PMC13016438; doi:10.2196/82997)
Supplement: Multimedia Appendix 9 [file jmir-v28-e82997-s009.docx]

# Multimedia Appendix 9

## Retrieval Augmented Generation Evaluation Results on German Dataset in Cross-Patient Setting with Answers Generated by Qwen-3.

The answers were generated with the *Qwen3-235B-A22B-Instruct-2507-FP8* LLM. Other parts of the RAG system were not changed to the original RAG-evaluation described in the manuscript. P stands for Precision and R stands for Recall.

| **Metrics** | multi-lingual-e5-large | miracle | miracle  pseudo-nymized | bge-m3 | gte-multi-lingual-base | german-bge-m3 |
| --- | --- | --- | --- | --- | --- | --- |
| **BERTScore P** | 0.748 | **0.765** | 0.758 | 0.745 | 0.739 | 0.746 |
| **BERTScore R** | 0.779 | **0.796** | 0.790 | 0.776 | 0.770 | 0.780 |
| **BERTScore F1** | 0.761 | **0.780** | 0.772 | 0.758 | 0.753 | 0.761 |
| **BLEURT** | 0.590 | **0.611** | 0.608 | 0.584 | 0.574 | 0.591 |
| **ROUGE-1** | 0.374 | **0.412** | 0.404 | 0.368 | 0.351 | 0.375 |
| **ROUGE-2** | 0.230 | **0.263** | 0.258 | 0.226 | 0.212 | 0.233 |
| **ROUGE-L** | 0.326 | **0.359** | 0.354 | 0.320 | 0.305 | 0.327 |
| **Contextual P** | 0.674 | 0.781 | **0.817** | 0.624 | 0.559 | 0.685 |
| **Contextual R** | 0.770 | 0.861 | **0.889** | 0.726 | 0.671 | 0.773 |
| **Contextual Relevancy** | 0.308 | **0.313** | 0.312 | 0.278 | 0.273 | 0.311 |
