# Supplementary material for: Improving Retrieval Augmented Generation for Health Care by Fine-Tuning Clinical Embedding Models: Development and Evaluation Study
Source: J Med Internet Res. 2026 Mar 25;28:e82997. doi: 10.2196/82997 (PMC13016438; doi:10.2196/82997)
Supplement: Multimedia Appendix 10 [file jmir-v28-e82997-s010.docx]

# Multimedia Appendix 10

## Retrieval Augmented Generation Evaluation Results on English Dataset in Cross-Patient Setting.

For the RAG evaluation, an RAG system was set up using the different embedding models as retrievers. With the help of different semantic evaluation metrics like BERTScore, BLEURT and ROUGE, the answer quality of the RAG system was evaluated. The dataset underlying the RAG system is a validation dataset that consists of real-world documents that were given as input to a LLM. The LLM generated questions and answer pairs based on these documents. The question and answer pairs were checked and filtered by a physician and then translated from German into English. The questions were asked to the RAG system and it had to find relevant document passages to answer the question. The generated answers were compared to the ground truth answers. P stands for Precision and R stands for Recall.

| **Metrics** | multilingual-e5-large | miracle-translated | miracle-translated  pseudonym-ized | bge-m3 | gte-multilingual-base |
| --- | --- | --- | --- | --- | --- |
| **BERTScore P** | 0.710 | 0.715 | **0.718** | **0.718** | **0.718** |
| **BERTScore R** | 0.774 | 0.784 | **0.788** | 0.779 | 0.777 |
| **BERTScore F1** | 0.740 | 0.747 | **0.750** | 0.746 | 0.746 |
| **BLEURT** | 0.475 | 0.490 | **0.497** | 0.475 | 0.474 |
| **ROUGE-1** | 0.289 | 0.305 | **0.313** | 0.305 | 0.305 |
| **ROUGE-2** | 0.129 | 0.141 | **0.146** | 0.137 | 0.136 |
| **ROUGE-L** | 0.235 | 0.249 | **0.254** | 0.248 | 0.247 |

# 
