# Supplementary material for: Improving Retrieval Augmented Generation for Health Care by Fine-Tuning Clinical Embedding Models: Development and Evaluation Study
Source: J Med Internet Res. 2026 Mar 25;28:e82997. doi: 10.2196/82997 (PMC13016438; doi:10.2196/82997)
Supplement: Multimedia Appendix 11 [file jmir-v28-e82997-s011.docx]

# Multimedia Appendix 11

## Retrieval Augmented Generation Evaluation Results on German Dataset in Patient-Centered Setting with Answers Generated by Qwen-3.

The answers were generated with the *Qwen3-235B-A22B-Instruct-2507-FP8* LLM. Other parts of the RAG system were not changed to the original RAG-evaluation described in the manuscript. P stands for Precision and R stands for Recall.

| **Metrics** | multi-lingual-e5-large | miracle | miracle  pseudo-nymized | bge-m3 | gte-multi-lingual-base | german-bge-m3 |
| --- | --- | --- | --- | --- | --- | --- |
| **BERTScore P** | 0.778 | **0.780** | **0.780** | 0.779 | 0.779 | 0.777 |
| **BERTScore R** | 0.800 | 0.802 | **0.803** | 0.800 | 0.800 | 0.800 |
| **BERTScore F1** | 0.787 | **0.789** | **0.789** | 0.787 | 0.786 | 0.786 |
| **BLEURT** | 0.646 | **0.649** | **0.649** | 0.647 | 0.644 | 0.643 |
| **ROUGE-1** | 0.451 | **0.456** | **0.456** | 0.453 | 0.450 | 0.448 |
| **ROUGE-2** | 0.296 | 0.300 | **0.301** | 0.297 | 0.293 | 0.293 |
| **ROUGE-L** | 0.398 | 0.402 | **0.403** | 0.401 | 0.397 | 0.395 |
| **Contextual P** | 0.872 | 0.919 | **0.932** | 0.864 | 0.826 | 0.881 |
| **Contextual R** | 0.930 | 0.939 | **0.940** | 0.935 | 0.914 | 0.930 |
| **Contextual Relevancy** | 0.239 | 0.241 | **0.248** | 0.239 | 0.235 | 0.241 |
