# Supplementary material for: Improving Retrieval Augmented Generation for Health Care by Fine-Tuning Clinical Embedding Models: Development and Evaluation Study
Source: J Med Internet Res. 2026 Mar 25;28:e82997. doi: 10.2196/82997 (PMC13016438; doi:10.2196/82997)

# Multimedia Appendix 12

## RAG Patient-Centered Evaluation Results per Document Category.

For the RAG evaluation, generated answers by the RAG system are compared to ground truth answers that were created by a Large Language Model and filtered by four human annotators. The higher the scores are, the more similar the answers are considered to the ground truth.

**B. Pathology reports**

**A. Discharge letters**


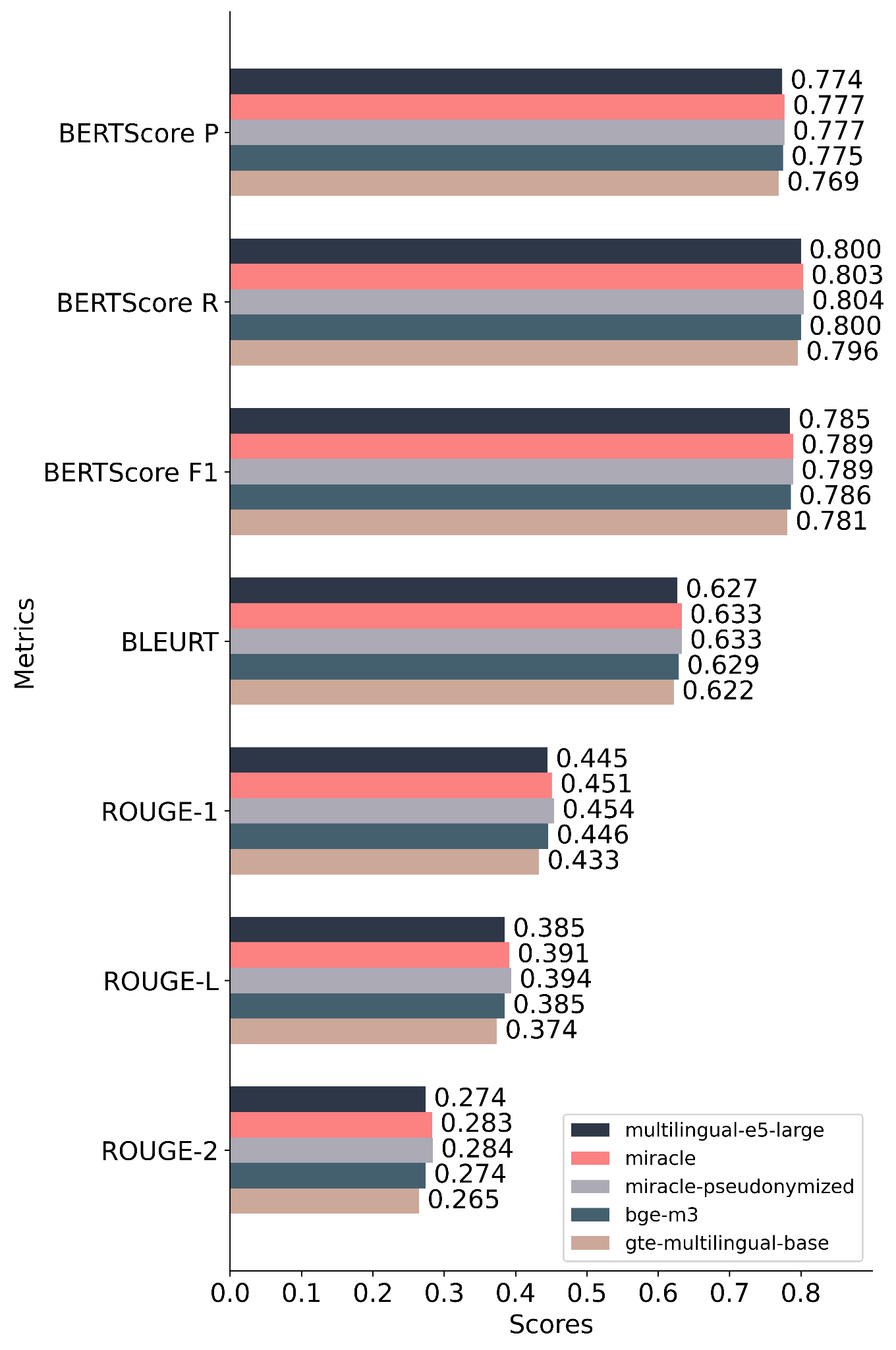

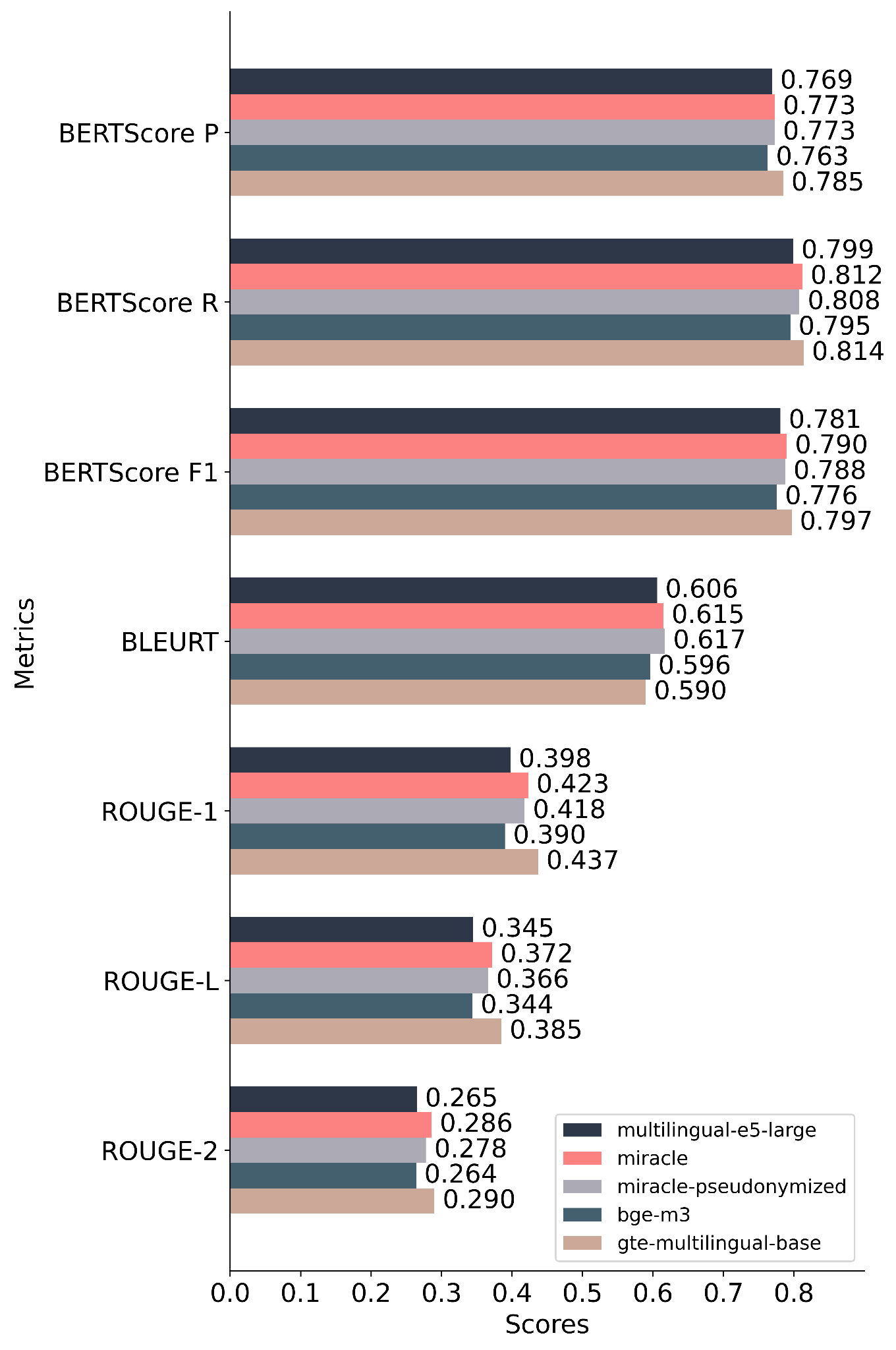


**D. Operation notes**

**C. Radiology reports**


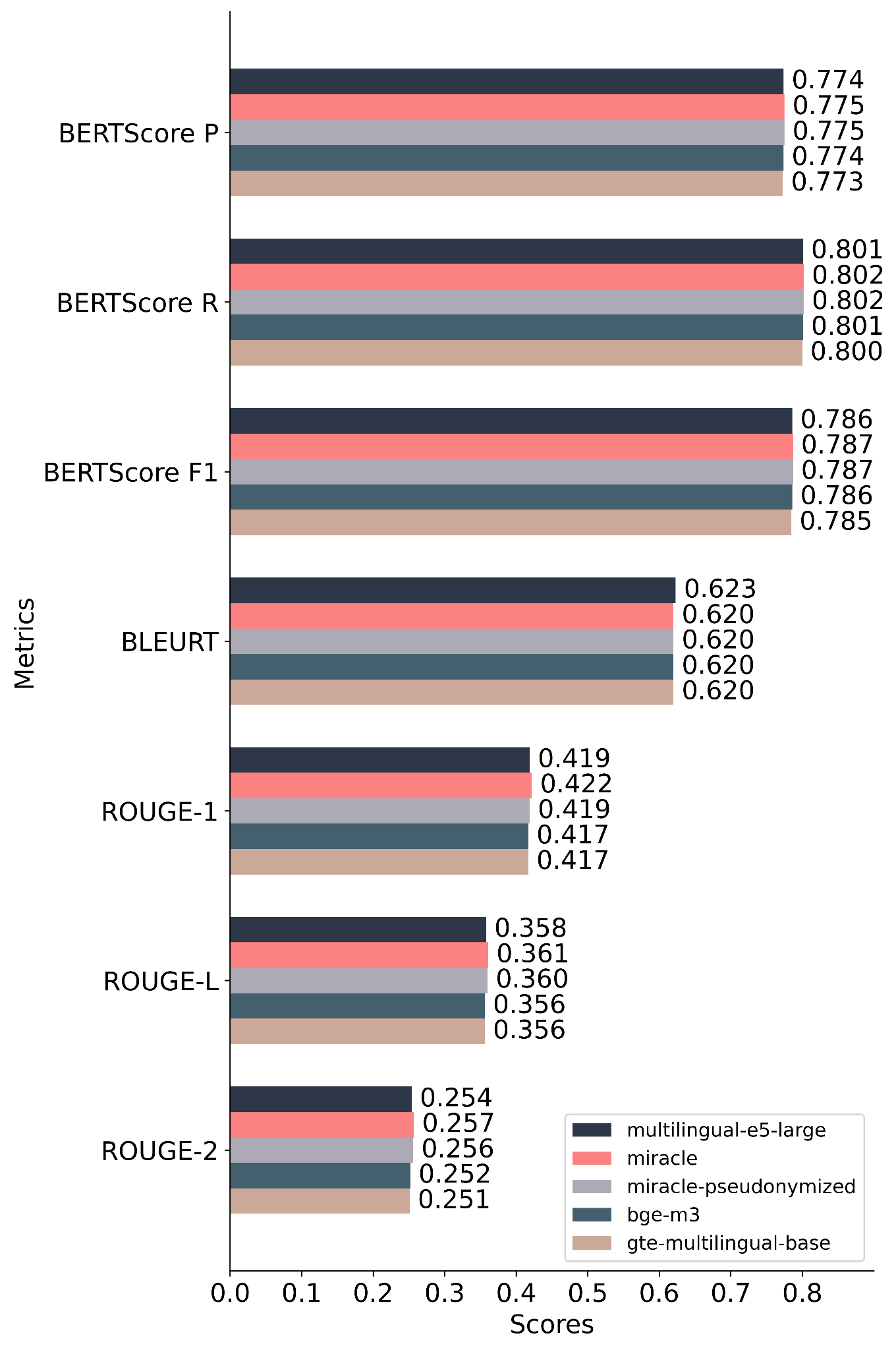

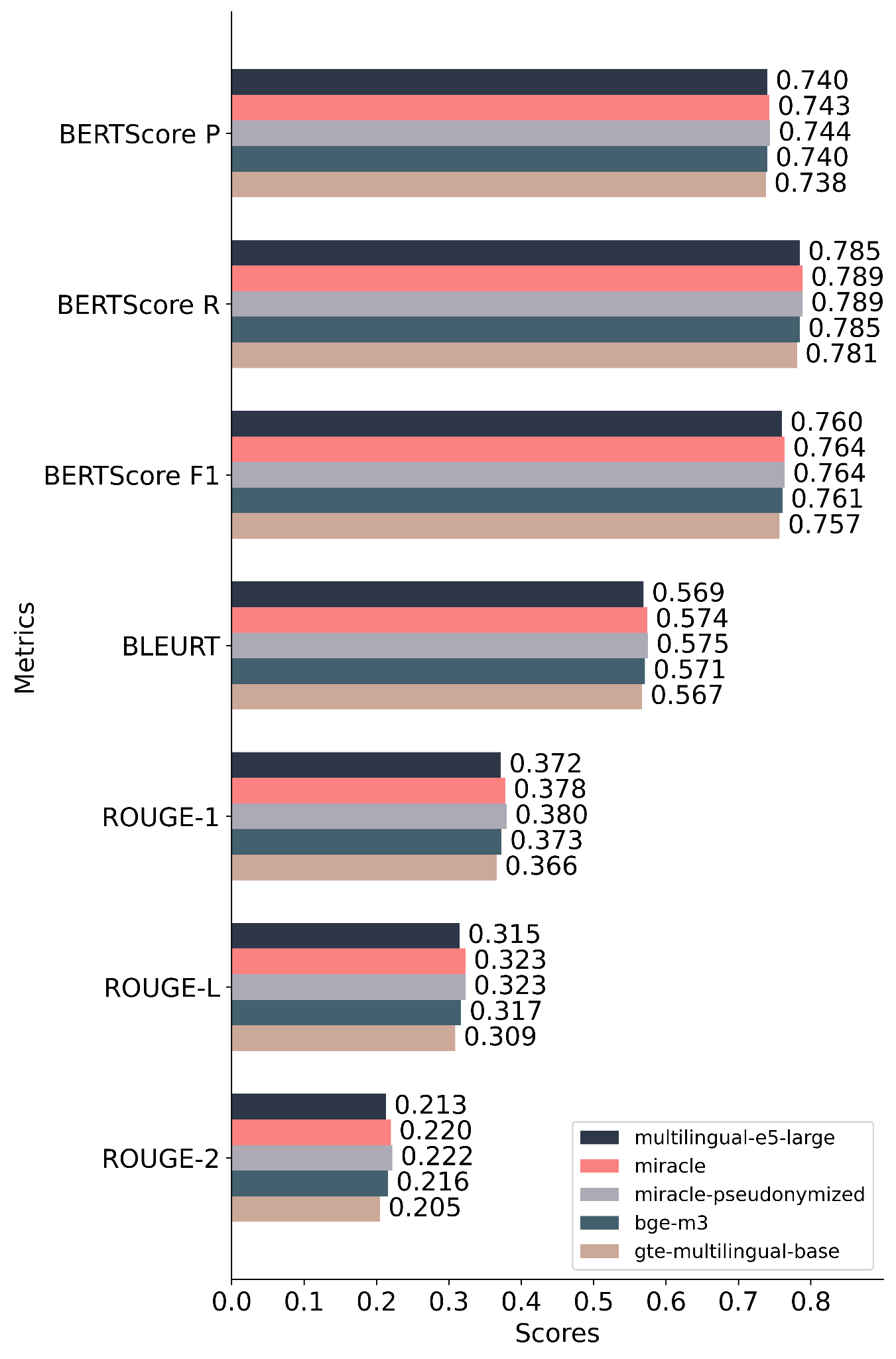

Supplement: Multimedia Appendix 12 [file jmir-v28-e82997-s012.docx]
