# Supplementary material for: Improving Retrieval Augmented Generation for Health Care by Fine-Tuning Clinical Embedding Models: Development and Evaluation Study
Source: J Med Internet Res. 2026 Mar 25;28:e82997. doi: 10.2196/82997 (PMC13016438; doi:10.2196/82997)

# Multimedia Appendix 13

## Mockup Implementation of Clinical RAG System with Integration into Electronic Health Record for Patient Question Answering.

The integration of the RAG system with domain and language-specific embedding models into Electronic Health Records could potentially speed-up information retrieval for physicians. This is a mock-up implementation to show what this integration would look like and how clinicians could use the embedding models to ask questions about a patient's medical history.


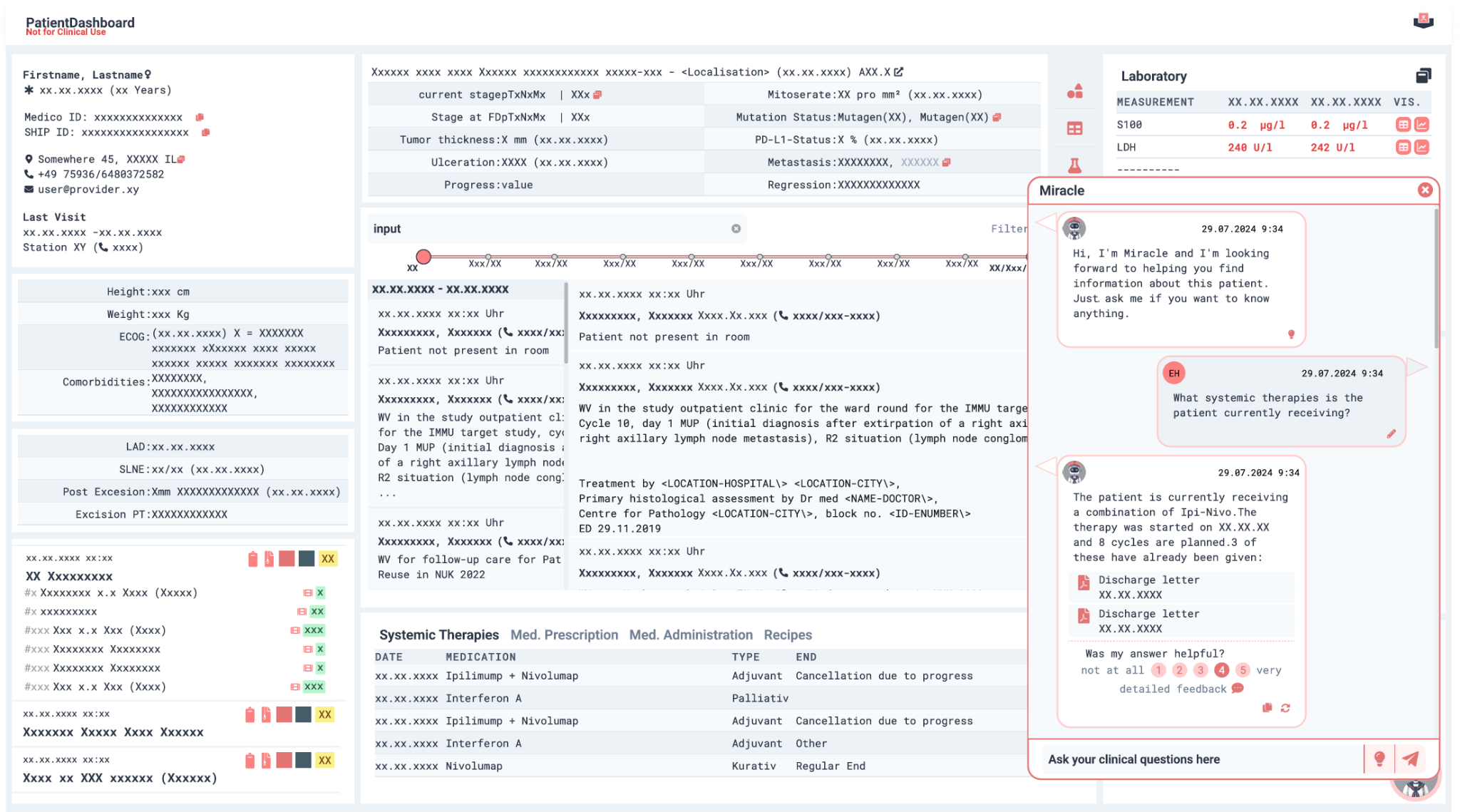

Supplement: Multimedia Appendix 13 [file jmir-v28-e82997-s013.docx]
